# Identification of a novel biomarker of antibody-mediated rejection in heart transplantation: synergistic effect of anti-nuclear antibodies and *de novo* donor-specific IgG HLA antibodies

**DOI:** 10.3389/fimmu.2025.1550779

**Published:** 2025-04-02

**Authors:** Maneesh Kumar Misra, Phillip McMullen, Gene H. Kim, Susana G. Marino

**Affiliations:** ^1^ Department of Pathology, Stanford University School of Medicine, Palo Alto, CA, United States; ^2^ Department of Pathology, The University of Chicago Medicine, Chicago, IL, United States; ^3^ Department of Pathology and Laboratory Medicine, Loyola University, Chicago, IL, United States; ^4^ Department of Cardiology, The University of Chicago Medicine, Chicago, IL, United States

**Keywords:** antibody mediated rejection, donor-specific antibodies, non-HLA antibodies, autoantibodies, heart transplant

## Abstract

**Introduction:**

Humoral autoimmune response may play a significant role in stimulating the alloimmune response, leading to antibody-mediated rejection (ABMR). This study investigated whether the development of IgG *de novo* donor-specific antibodies (dnDSA) could serve as an independent marker for ABMR diagnosis. Subsequently, we evaluated the synergistic effects of non-HLA anti-nuclear antibodies (ANA) and circulating IgG anti-HLA dnDSA in the development of ABMR.

**Methods:**

This retrospective study included 285 patients who underwent heart transplants between January 2007 to November 2020 at the University of Chicago Medical Center and who had sufficient serum collected at the time of protocol or indication biopsy available for antibody testing.

**Results:**

We observed a 23% incidence of ABMR in heart transplant patients at our center. Kaplan–Meier survival analysis revealed the lowest ABMR free survival in recipients that were positive for both ANA and circulating IgG dnDSA (Log rank p = 2 x 10^-16^), indicating a synergistic effect of ANA and circulating IgG dnDSA. A univariate stepwise cox proportional hazard model establishes the presence of IgG dnDSA as an independent marker to predict ABMR diagnosis (HR = 8.70, p = 6.15 x 10^-9^). Similarly, a synergistic effect was found in the presence of a positive ANA titer and IgG dnDSA for ABMR diagnosis in a univariate model (HR = 13.1, p = 2.73 x 10^-14^). A multivariate stepwise cox proportional hazard model showed an almost seven-fold increased risk for ABMR in patients that have developed IgG dnDSA (HR = 6.96, p = 2.33 x 10^-6^). Similarly, nearly an eleven-fold enhanced risk for ABMR was found in heart transplant recipients who were positive for ANA and had developed *de novo* IgG DSA (HR = 10.7, p = 1.25 x 10^-10^), suggesting the synergistic effect of ANA and IgG dnDSA in ABMR diagnosis.

**Discussion:**

This study establishes circulating IgG dnDSA as an independent biomarker for ABMR diagnosis in heart transplantation and confirms the previously known correlation of IgG dnDSA with ABMR. Subsequently, our data revealed that circulating IgG dnDSA and non-HLA antinuclear antibodies have synergistic effects that cause antibody-mediated rejection in heart transplantation.

## Introduction

Heart transplant (HTx) remains the treatment-of-choice for end-stage cardiac disease (1). Antibody-mediated rejection (ABMR), driven by the development of *de novo* donor-specific antibodies (dnDSA) directed against mismatched donor human leukocyte antigen (HLA), is a significant risk factor for graft loss in cardiac transplantation (2). Further, the antibodies against non-HLA autoantigens are also linked with poor graft survival in solid organ transplant (3, 4). The clinically significant non-HLA antibodies are of two types, first the alloantibodies directed against mismatched polymorphic donor antigens (5), and second, the antibodies that target self-antigens are referred to as autoantibodies (4, 6). Accumulating evidence indicates that the humoral autoimmune response may play a significant role in both acute and chronic transplant rejection (7–9). These studies noted the development of anti-endothelial and cardiac tissue-specific IgG antibodies, which bind both to self and allo-determinants (8, 9). The breakdown in B cell tolerance to self is complex. The humoral autoimmunity described in human transplant recipients has diverse patterns. It seems likely that different processes govern its generation in different patients or for different organs. Recent studies revealed that the target autoantigens are expressed specifically in the donor organ, such as vimentin intermediate filament (4), myosin motor protein (10, 11), and skeletal muscle glycolipid (12) in post-heart transplant recipients, glutamic acid decarboxylase (GAD) enzyme and islet antigens in post-transplant pancreas patients (13), and glomerular basement membrane protein agrin in renal transplant recipients (14). It is still unknown whether these different processes impact allograft outcomes to the same extent. Therefore, this is an important area for research that can provide information to better develop early therapeutic strategies that target humoral autoimmunity to prevent graft rejection and graft failure.

Several studies have indicated that antibodies to non-HLA antigens can also mediate ABMR, including autoantibodies generated by the solid organ transplant recipient (3, 15, 16). In 1987, Graham and colleagues demonstrated that positive antinuclear antibody (ANA) titers (greater than 1:160) in patients undergoing cardiac transplantation have an increased risk of mortality (17). Further, high ANA titers have been associated with the progression of atherosclerosis in non-transplant cardiac patients (18), and a role of autoantibodies in the etiology of coronary artery disease has been suggested (19). Our group has recently reported that the presence of clinically significant ANA titers in patients awaiting heart transplantation is associated with increased mortality (20). Similarly, a recent study suggests that humoral autoimmune response may significantly contribute to acute and chronic solid organ transplant rejection (7). Therefore, in the present study, we hypothesize that an underlying humoral autoimmune condition could trigger or contribute to the development of IgG dnDSA directed against the mismatched donor HLA antigens and, thus, either initiate the development of *de novo* alloimmune response or could synergistically with *de novo* alloimmune response cause the development of antibody-mediated rejection in cardiac transplantation.

This study aimed to determine the role of the development of dnDSA in predicting the ABMR diagnosis. Subsequently, we investigated a correlation between ANA titers, a novel biomarker of autoimmune condition, with the development of dnDSA directed against the mismatched donor HLA antigens in cardiac transplant patients and evaluated the synergistic effect of ANA and IgG dnDSA in ABMR diagnosis.

## Material and methods

### Patients and clinical data

This retrospective study included a combined cohort of 285 patients who underwent HTx between January 2007 and November 2020 at the University of Chicago Medical Center and who had sufficient serum collected at the time of protocol or indication biopsy available for antibody testing. The data for the discovery cohort was collected from 113 patients transplanted between January 2007 to July 2012, and the replication cohort includes 172 cases who received heart transplants between October 2012 to November 2020. The endomyocardial biopsies were scored according to the 2013 or 2004 International Society for Heart and Lung Transplantation criteria for ABMR. All the heart transplant recipients receive either basiliximab or antithymocyte globulin induction therapy based on immunological risk and a calcineurin inhibitor-based immunosuppressive regimen with mycophenolate mofetil and tapering doses of corticosteroids during the first year after transplantation. This study was reviewed and approved by the University of Chicago, Institution Review Board (IRB 22-0356).

### Antinuclear antibody testing

Anti-nuclear antibody testing was performed by indirect immunofluorescence (IFA) using EUROIMMUN IFA 40: HEp-20-10 EUROPattern assay according to the manufacturer’s instructions. This test allows *in vitro* detection of anti-IgG nuclear antibodies in human serum with a EUROPattern Microscope and Software automated instrument. An ANA titer of ≥ 1:160 was considered positive.

### HLA antibody testing

Serum samples were screened for anti-HLA IgG antibodies using the standard protocol for LABScreen mixed beads (ONE LAMBDA, Canoga Park, CA). If positive, the specificities were characterized using LABScreen Single Antigen beads (ONE LAMBDA, SAB, Canoga Park, CA) according to the manufacturer’s instructions. Data was acquired on the LABScan 200, and results were analyzed using HLA FUSION software (One Lambda). For a serum with a high background, an adsorption step with Luminex beads without antigen coating was performed to remove nonspecific binding. The anti-HLA antibody positivity was determined by antibody pattern analysis on both LABScreen mixed beads and SAB assays, and antibody assignment was made with a baseline MFI cutoff of ≥ 250 on LABScreen mixed beads and ≥ 750 on LABScreen SAB assays. A combination of LABScreen mixed beads (natural beads) and SAB assays was used in specificity assignments to avoid the antibody calling against denatured antigens. Post-transplant circulating IgG DSA monitoring was performed on months 1, 3, 6, and 12 in the first year, every three months in years 2 and 3, and every 6 months thereafter. Post-transplant HLA IgG DSA testing was performed on the same post-transplant sera collected at the time of protocol or indication endomyocardial biopsy.

### Statistical analysis

Descriptive statistics were used to summarize the baseline and demographics. Qualitative data were expressed as frequency and percentage, whereas the continuous variables were represented as means and standard deviations. Survival analysis was performed by the Kaplan‐Meier method using the log‐rank test for significance. Univariate and multivariate Cox proportional hazard regression models were used to estimate the crude and adjusted hazard ratios (HRs) and their 95% CIs. Statistical significance was considered at p ≤ 0.05. Statistical analysis was performed using the R language for statistical computing (https://cran.r-project.org/bin/windows/base/old/4.2.2/).

## Results

### Demographics characteristics

During the study period, 285 patients underwent heart transplantation at our center. Our discovery and validation cohorts consist of 113 and 172 heart transplant patients, respectively. The patient characteristics and demographic profiles of discovery, validation, and combined cohort are presented in **Table 1**.

**Table 1:** Patient demographics characteristics.

Characteristics	Discover Cohort (n = 113)	Validation Cohort (n = 172)	Combined Cohort (n = 285)
Male n (%)	88 (78%)	134 (78%)	222 (78%)
Age at transplant (Mean ± SD)	51 ± 14	53 ± 14	52 ± 14
Etiology of Heart Failure			
ICM	44 (38.94%)	45 (26.16%)	89 (31.12%)
NICM	69 (61.06 %)	127 (73.84%)	196 (68.53%)
Race, n (%)			
Caucasian	66 (58.41%)	111 (64.53%)	177 (61.89%)
African American	38 (33.63%)	44 (25.58%)	82 (28.69%)
Hispanic	4 (3.54%)	6 (3.49%)	10 (3.50%)
East Asian	4 (3.54%)	3 (1.74%)	7 (2.45%)
Middle East	1 (0.88%)	8 (4.65%	9 (3.15%)
De novo DSA(s)	14 (12%)	20 (12%)	34 (12%)
ABMR n (%)	20 (18%)	46 (27%)	66 (23%)
HLA-A, B, DR Antigen Mismatch (Mean ± SD)	5.12 ± 0.96	5.05 ± 0.99	5.08 ± 0.98

### Incidence of ABMR and correlation of development of IgG dnDSA with ABMR

**Figure 1:**
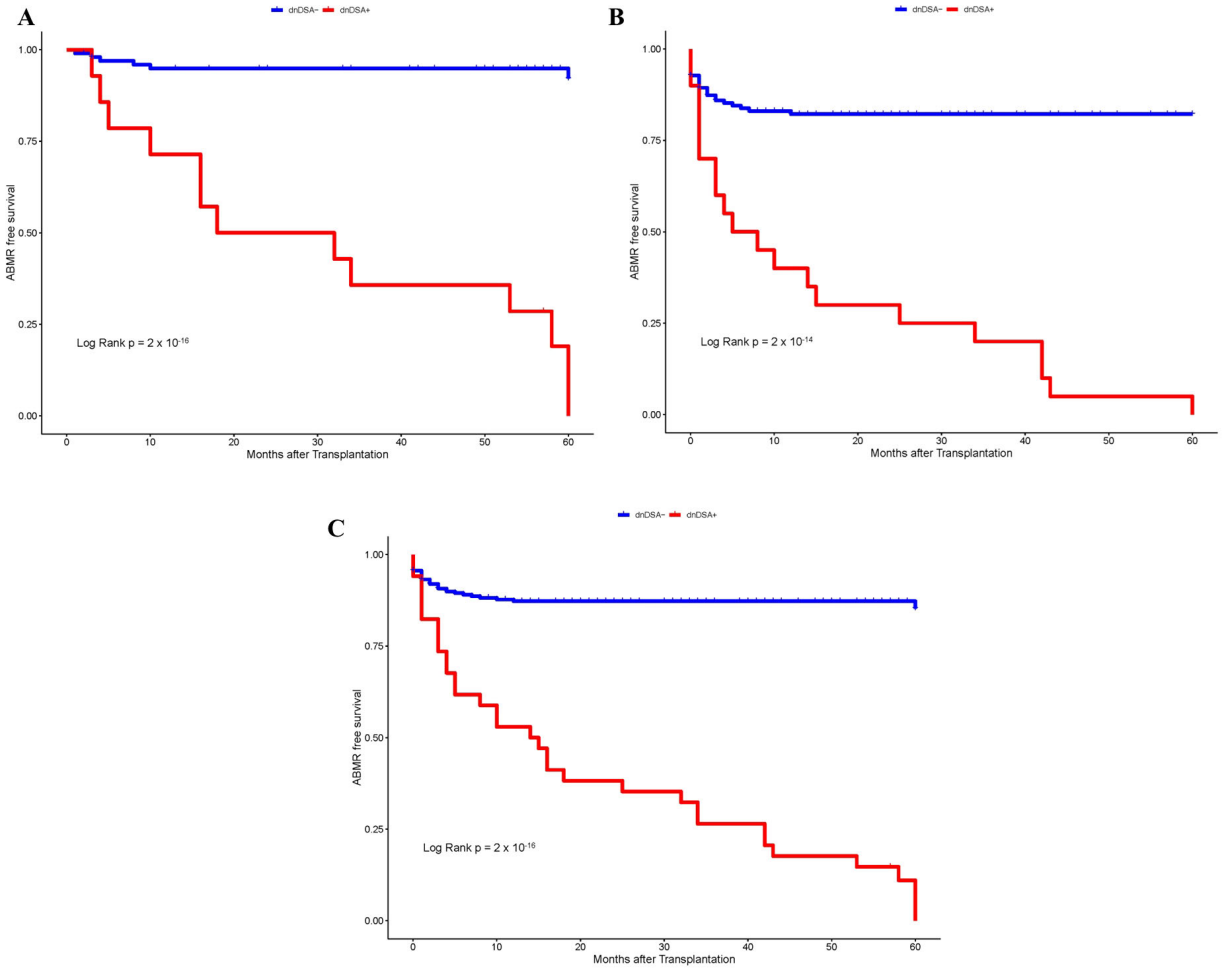
Kaplan–Meier survival analysis of ABMR free survival by IgG dnDSA status. **(A)** Discovery Cohort. **(B)** Validation Cohort. **(C)** Combined Cohort.

First, we investigated whether the development of dnDSA can serve as an independent biomarker for the diagnosis of ABMR. Overall, the incidence of ABMR was 23% at our center, which ranged from 18% to 27% in the discovery and validation cohorts, respectively (**Table 1**). The higher prevalence of ABMR in the validation cohort (27%) as compared to the discovery cohort (18%) could most likely be attributed to closer monitoring of post-transplant in the replication cohort by IgG DSA(s) testing and endomyocardial protocol biopsies. The prevalence of IgG dnDSA remained stable at 12% in the combined discovery and validation cohort. The heart transplant recipients included in the present study were followed for up to 5 years. Kaplan–Meier survival analysis was first performed to assess the probability of ABMR-free survival according to post-Tx IgG dnDSA status to determine their significance in our heart transplant patient population. Interestingly, we found a significant correlation between the development of IgG dnDSA and ABMR in the discovery cohort (Log-rank p = 2 x 10^-16^, **Figure 1A**), and the finding in the discovery cohort was replicated in the validation cohort (Log-rank p = 2 x 10^-14^, **Figure 1B**), and these observations were consistent in the combined cohort as well (Log rank p = 2 x 10^-16^, **Figure 1C**). Subsequently, we observed that the presence of IgG dnDSA against mismatch donor HLA antigens was independently associated with ABMR rejection in the discovery (OR = 27.3, p = 6.98 x 10^-11^) cohort and the finding was replicated in the validation cohort (OR = 7.14, p = 4.7 x 10^-11^), and were found consistent in combined cohort as well (OR = 10.3, p = 2.2 x 10^-16^), establishing IgG DSA as an independent biomarker to predict ABMR in heart transplant patients.

### Assessment of synergistic effect of ANA titer and IgG dnDSA(s) to predict ABMR

**Figure 2:**
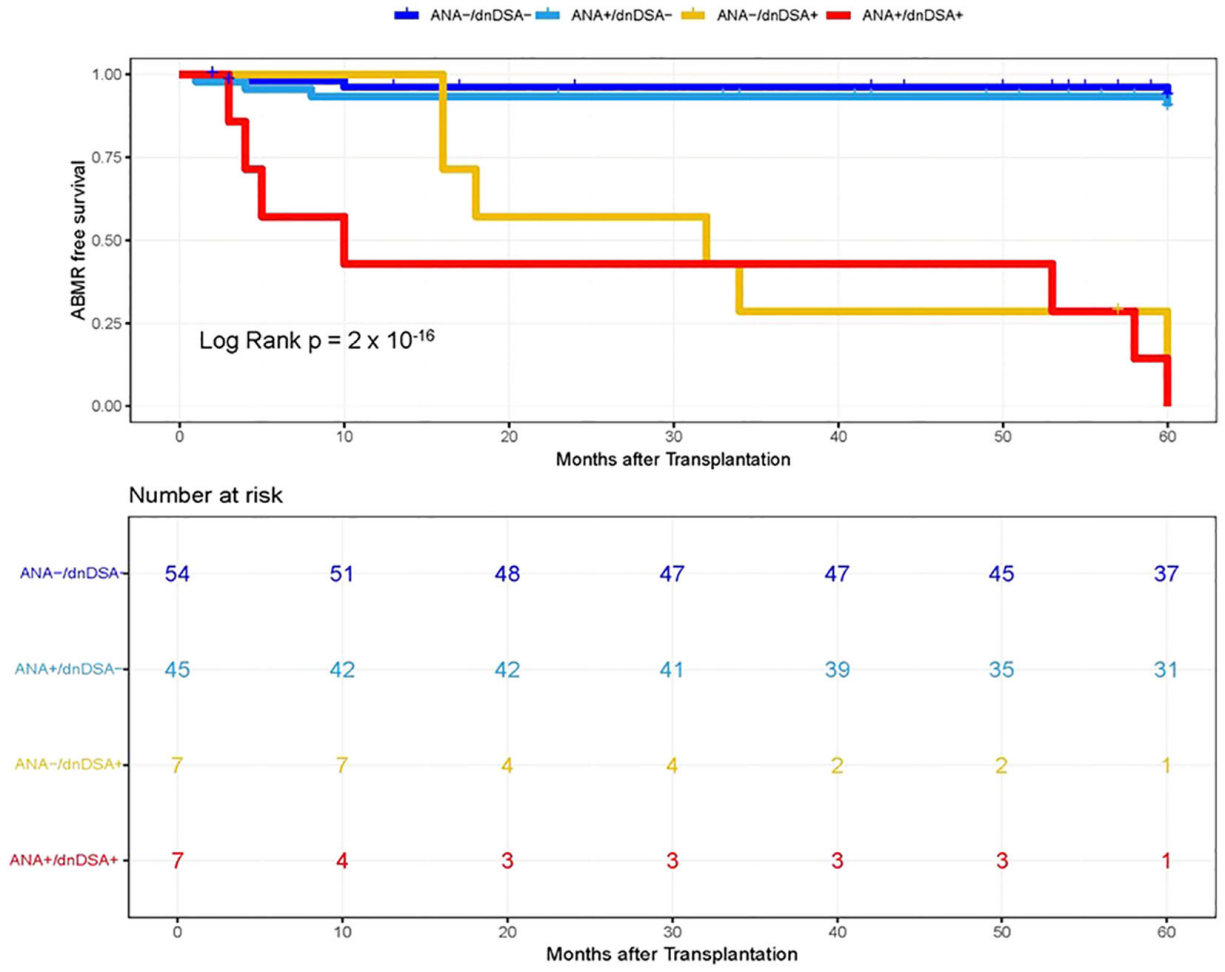
Kaplan–Meier survival analysis of ABMR free survival by ANA and IgG dnDSA status in Discovery Cohort. Heart transplant recipients were categorized into four groups namely, ANA-/dnDSA-, ANA negative/dnDSA negative; ANA+/dnDSA-, ANA positive/dnDSA Negative; ANA-/dnDSA+ = ANA Negative/dnDSA positive; ANA+/dnDSA+ = ANA positive/dnDSA positive.

**Figure 3:**
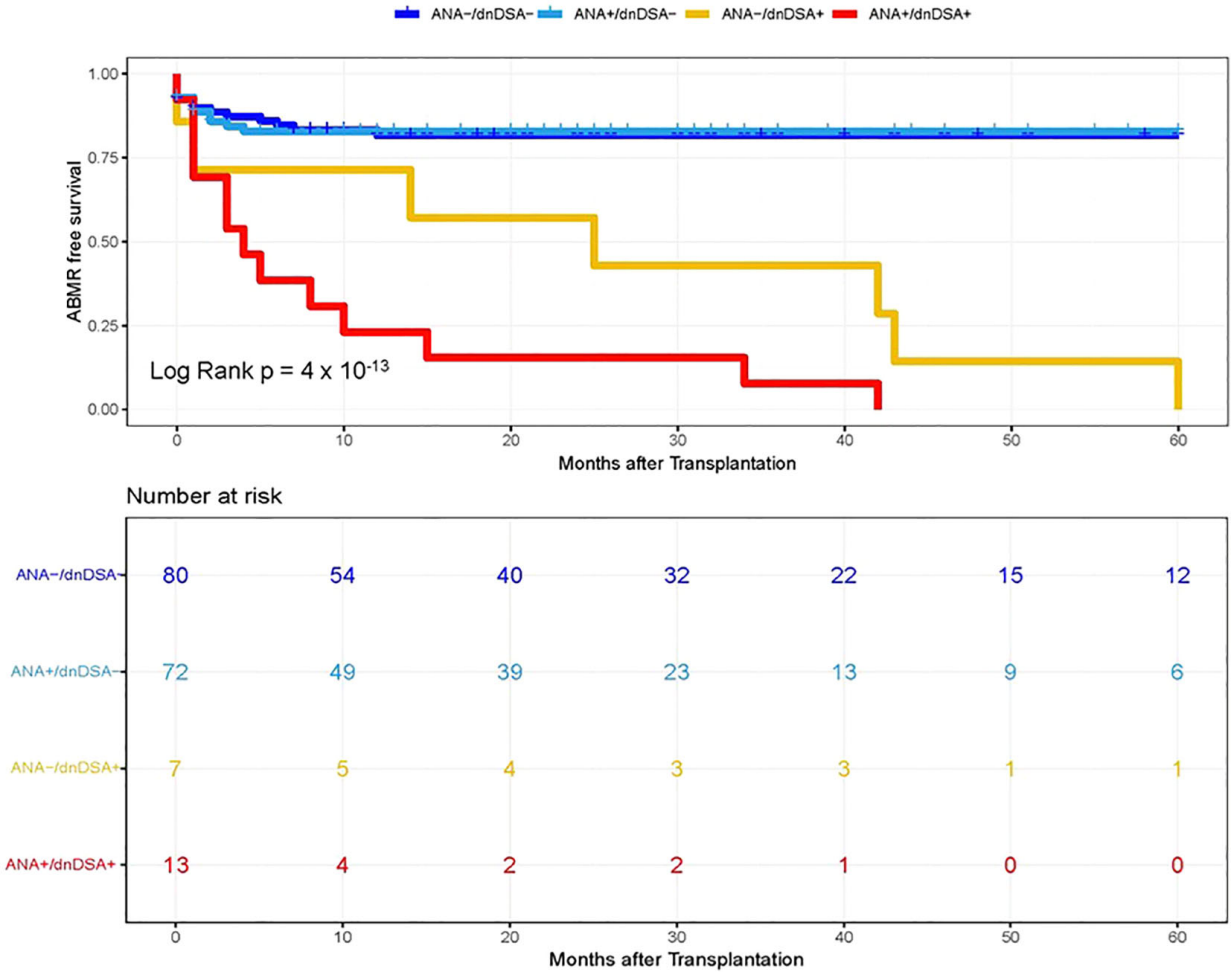
Kaplan–Meier survival analysis of ABMR free survival by ANA and IgG dnDSA status in Validation Cohort. Heart transplant recipients were categorized into four groups namely, ANA-/dnDSA-, ANA negative/dnDSA negative; ANA+/dnDSA-, ANA positive/dnDSA Negative; ANA-/dnDSA+ = ANA Negative/dnDSA positive; ANA+/dnDSA+ = ANA positive/dnDSA positive.

**Figure 4:**
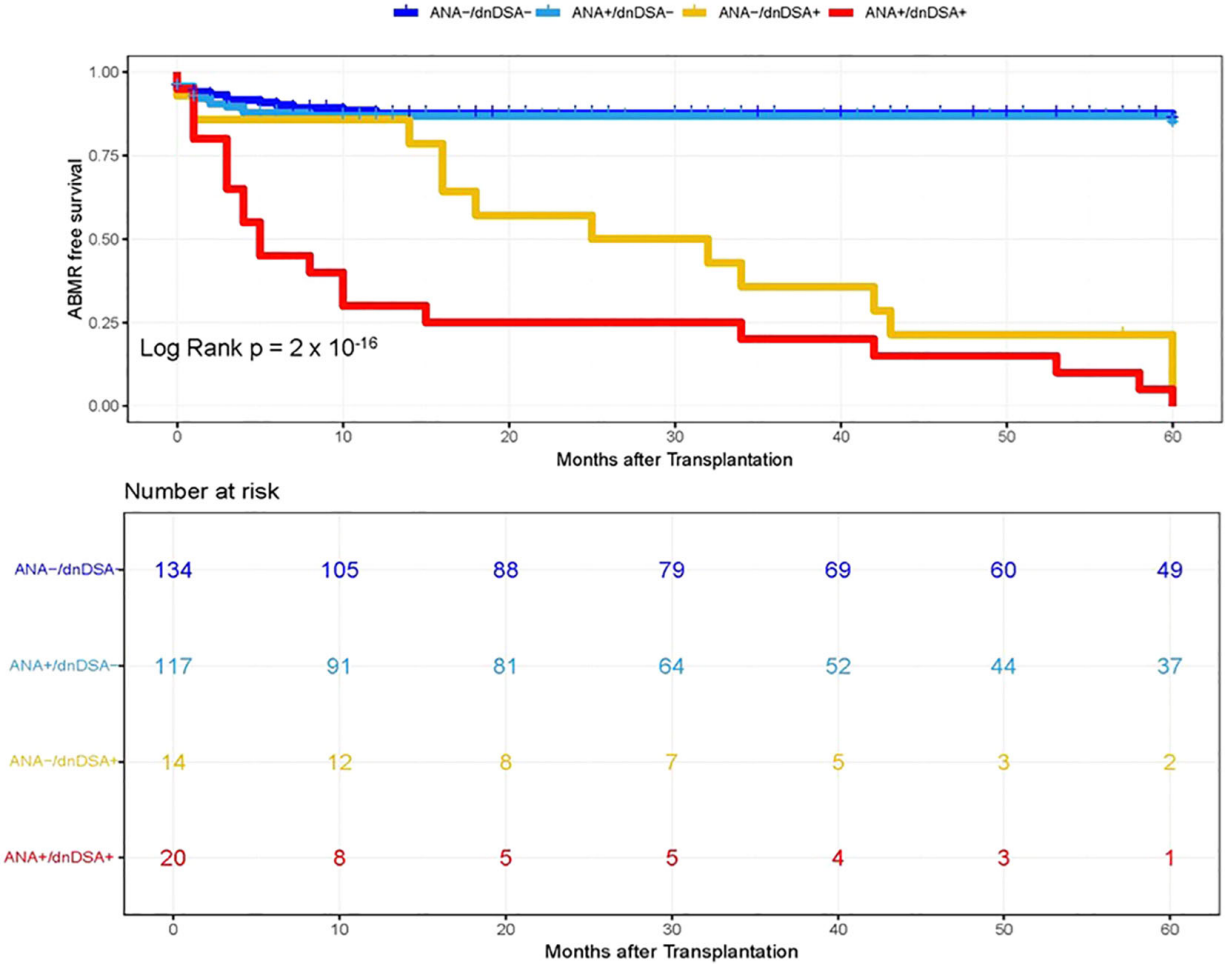
Kaplan–Meier survival analysis of ABMR free survival by ANA and IgG dnDSA status in Combined Cohort. Heart transplant recipients were categorized into four groups namely, ANA-/dnDSA-, ANA negative/dnDSA negative; ANA+/dnDSA-, ANA positive/dnDSA Negative; ANA-/dnDSA+ = ANA Negative/dnDSA positive; ANA+/dnDSA+ = ANA positive/dnDSA positive.

In the next step, we investigated whether pre-transplant ANA titers are associated with the development of dnDSA and assessed the synergistic effects of ANA and dnDSA with ABMR diagnosis. For this purpose, we categorize the patients into four groups based on the ANA titer and presence of IgG dnDSA, namely, ANA negative/dnDSA negative, ANA positive/dnDSA Negative, ANA Negative/dnDSA positive, and ANA positive/dnDSA positive. Kaplan–Meier survival analysis was performed according to the ANA and IgG DSA status. We observed the lowest ABMR free survival in recipients that were positive for both ANA and IgG dnDSA in the discovery cohort (Log-rank p = 2 x 10^-16^, **Figure 2**), indicating a synergistic effect of ANA and IgG dnDSA in the development of ABMR. This observation was replicated in the validation cohort (Log-rank p = 4 x 10^-13^, **Figure 3**). Subsequently, a combined analysis of datasets from the discovery and validation cohort further establishes and refines the role of synergistic effects of ANA and IgG dnDSA by demonstrating the poorest ABMR free survival in recipients positive for both ANA and circulating IgG dnDSA (Log-rank p = 2 x 10^-16^, **Figure 4**). Interestingly, the combined dataset showed a good correlation between ANA titer and dnDSA status with ABMR-free survival. Since the combined dataset was larger, therefore, we can see a good separation in survival curves between the ANA Negative/dnDSA positive and ANA positive/dnDSA positive cases (**Figure 4**). We observed ~62.5% lower survival in ANA Negative/dnDSA positive cases than ANA negative/dnDSA negative cases. Interestingly, we observed the lowest survival in heart transplant recipients that were positive for both ANA and IgG dnDSA and a survival difference of ~16% with reference to cases that were IgG DSA positive but negative for ANA, suggesting the synergistic effect of ANA and IgG dnDSA (Log rank p = 2 x 10^-16^, **Figure 4**).

An analysis under the univariate stepwise cox proportional hazard model establishes the presence of IgG dnDSA as an independent marker to predict ABMR in the discovery cohort (HR = 28.4, p = 5.59 x 10^-6^, **Table 2**), and this observation was validated in replication cohort (HR = 5.81, p = 1.49 x 10^-4^, **Table 2**). Similarly, a synergistic effect was seen in the presence of positive ANA titer and the presence of IgG dnDSA to predict the ABMR in the discovery cohort (HR = 42.1, p = 1.63 x 10^-7^, **Table 2**), and the finding was consistent in replication cohort (HR = 8.36, p = 6.13 x 10^-8^, **Table 2**). Subsequently, an analysis was performed after combining the discovery and replication cohort dataset to increase the sample size and further demonstrate and reiterate that the presence of IgG dnDSA(s) served as an independent marker to predict ABMR-free survival under univariate stepwise Cox proportional hazard model (HR = 8.70, p = 6.15 x 10^-9^, **Table 2**). Likewise, a synergistic effect was noticed in the presence of a positive ANA titer and IgG dnDSA to predict ABMR-free survival in a combined cohort (HR = 13.1, p = 2.73 x 10^-14^, **Table 2**).

**Table 2:** Univariate model for assessment of synergistic effect of ANA titer and circulating IgG dnDSA(s) to predict ABMR in heart transplantation.

Characteristics	Discover Cohort (n = 113)			Validation Cohort (n = 172)			Combined Cohort (n = 285)		
ANA-DSA Status	HR	95% CI	p-Value	HR	95% CI	p-Value	HR	95% CI	p-Value
0	─	─		─	─		─	─	
1	1.59	0.36 - 7.09	0.545	1.02	0.47 – 2.22	0.952	1.13	0.57 – 2.24	0.718
2	28.4	6.70 - 120	5.59 x 10^-6*^	5.81	2.34 – 14.4	1.49 x 10^-4*^	8.70	4.20 – 18.1	6.15 x 10^-9*^
3	42.1	10.4 - 171	1.63 x 10^-7*^	8.36	3.88 – 18.0	6.13 x 10^-8*^	13.1	6.75 – 25.4	2.73 x 10^-14*^

0 = ANA─/DSA─; 1 = ANA+/DSA─; 2 = ANA─/DSA+; 3 = ANA+/DSA+; HR, Hazard Ratio; CI, Confidence Interval; *Statistically significant p -value.

Finally, a multivariate stepwise Cox proportional hazard model was used to evaluate the correlation between variables, including selected demographic characteristics (age, sex, and race), patient diagnosis, HLA-A, B, DRB1 mismatches, ANA and IgG dnDSA status, and ABMR free survival. Interestingly, we observed an almost seven-fold increased risk for ABMR rejections in patients that have developed IgG dnDSA (HR = 6.96, p = 2.33 x 10^-6^, **Table 3**), establishing the presence of IgG dnDSA as an independent marker to predict ABMR rejection. Similarly, nearly an eleven-fold enhanced risk for ABMR rejection was found in heart transplant recipients that were positive for ANA and had developed *de novo* IgG DSA (HR = 10.7, p = 1.25 x 10^-10^, **Table 2**), suggesting the synergistic effect of ANA and IgG dnDSA.

**Table 3:** Multivariate model for assessment of synergistic effect of ANA titer and circulating IgG dnDSA(s) to predict ABMR in heart transplantation.

Characteristics	Combined Cohort (n = 285)		
ANA-DSA Status	HR	95% CI	p-Value
0	─	─	
1	1.02	0.50 – 2.06	0.957
2	6.96	3.11 – 15.6	2.33 x 10^-6*^
3	10.7	5.19 – 22.0	1.25 x 10^-10*^
Age	0.99	0.97 – 1.01	0.263
Sex	0.97	0.54 – 1.75	0.930
Race	0.86	0.59 – 1.27	0.459
Diagnosis	0.89	0.48 – 1.64	0.712
ABDR Mismatches	1.22	0.69 – 2.14	0.497

0 = ANA─/DSA─; 1 = ANA+/DSA─; 2 = ANA─/DSA+; 3 = ANA+/DSA+; HR, Hazard Ratio; CI, Confidence Interval; *Statistically significant p -value.

## Discussion

Identification of noninvasive biomarkers to predict heart allograft outcomes and optimize risk stratification of long-term allograft failure at the individual patient level is a primary goal for the scientific community of heart transplant professionals. ABMR of allogenic heart transplants is inadequately defined and is considered a challenging diagnosis for allograft recipients and their physicians. Although the correlation between the presence of a detectable level of HLA DSA and ABMR is strong (21, 22), it is imperfect in heart transplant recipients, considering the reports of ABMR diagnosis in the absence of HLA DSA (4, 6, 16). The current gold standard for ABMR diagnosis is endomyocardial biopsy (EMB). However, the EMB alone may not be conclusive, as the disease may be focal (16). At the present time, circulating DSA is not considered a standard diagnostic, although it is under active consideration for inclusion in the ABMR diagnostic criteria within both the Banff (23) and International Society for Heart and Lung Transplantation (ISHLT) classifications for heart transplantation (24). Because histologic/pathologic ABMR can occur even in the absence of detectable levels of circulating DSAs, it may be potentially related to the presence of non-HLA antibodies (4–6, 25). Therefore, there is a need to refine the role of HLA DSA in ABMR diagnosis and actively search for other invasive and non-invasive non-HLA related biomarkers for ABMR diagnosis. In the present study, we investigated whether the development of IgG dnDSA could serve as an independent marker for ABMR diagnosis. Subsequently, we assessed the synergistic effects of non-HLA anti-nuclear antibodies and circulating anti-HLA IgG dnDSA in predicting ABMR diagnosis.

At our center, we observed 23% incidence of ABMR in heart transplant patients, which ranged from 18% to 27% in the discovery and validation cohort, respectively (**Table 1**). In previous reports, the prevalence of ABMR ranged from 17-30% in heart transplant recipients (21, 26, 27), which is consistent with our findings presented in this study. The heart transplant recipients included in the present study were followed for up to 5 years. Kaplan–Meier survival analysis revealed a significant correlation between the development of dnDSA and ABMR-free survival combined cohort (Log rank p = 2 x 10^-16^, **Figure 1**). Subsequently, we observed that the presence of IgG dnDSA was independently associated with ABMR diagnosis (OR = 10.3, p = 2.2 x 10^-16^), establishing IgG DSA as an independent biomarker to predict ABMR diagnosis in heart transplants. These observations were consistent with findings in the discovery and validation cohorts (**Figure 1**, **Table 2**). Likewise, previous studies reported a correlation of IgG dnDSA with ABMR (21, 22, 26, 27), further strengthening our observations. On the contrary, some studies have reported the incidence of ABMR in the absence of detectable levels of IgG DSA (25, 28).

Recent studies suggest that the humoral autoimmune response may play a significant role in the stimulation of the alloimmune response, leading to heart transplant rejection (7–9). Therefore, in the next step, we investigated a correlation between ANA titers, a novel biomarker of an autoimmune condition, with the development of dnDSA directed against the mismatched donor HLA antigens in cardiac transplant patients and assessed the synergistic effects of ANA and circulating IgG dnDSA with ABMR diagnosis. We observed the lowest ABMR free survival in recipients positive for both ANA and circulating IgG dnDSA, suggesting a synergistic effect of ANA and IgG dnDSA in the development of ABMR (Log rank p = 2 x 10^-16^, **Figure 4**). In addition, we observed ~62.5% lower survival in ANA Negative/dnDSA positive cases as compared to ANA negative/dnDSA negative cases. Interestingly, we observed the lowest survival in heart transplant recipients that were positive for both ANA and IgG dnDSA and a survival difference of ~16% with reference to cases that were IgG DSA positive but negative for ANA, suggesting the synergistic effect of ANA and IgG dnDSA (Log-rank p = 2 x 10^-16^, **Figure 4**). A univariate stepwise cox proportional hazard model establishes the presence of IgG dnDSA as an independent marker to predict ABMR diagnosis in a combined cohort (HR = 8.70, p = 6.15 x 10^-9^, **Table 2**). Similarly, a synergistic effect was found in the presence of a positive ANA titer and IgG dnDSA to predict the ABMR diagnosis in a combined cohort under a univariate model (HR = 13.1, p = 2.73 x 10^-14^, **Table 2**). A multivariate stepwise Cox proportional hazard model showed an almost seven-fold increased risk for ABMR in patients that have developed IgG dnDSA (HR = 6.96, p = 2.33 x 10^-6^, **Table 3**), establishing the presence of the IgG dnDSA as an independent marker to predict ABMR diagnosis. Similarly, nearly an eleven-fold enhanced risk for ABMR was found in heart transplant recipients who were positive for ANA and had developed *de novo* IgG DSA (HR = 10.7, p = 1.25 x 10^-10^, **Table 2**), suggesting the synergistic effect of ANA and IgG dnDSA in ABMR diagnosis. To our knowledge, this is the first study that evaluated the synergistic effect of ANA and IgG dnDSA on ABMR diagnosis. However, a previous report suggested an increased risk of mortality in heart transplant patients with positive ANA titers (greater than 1:160) (17), which was consistent with our observation of the synergistic effect of ANA and IgG dnDSA in decreased ABMR-free survival (**Figure 4**). Similar studies have shown the synergistic effect of non-HLA and IgG DSA in promoting allograft rejection (29, 30), further strengthening our findings presented in this study.

**Figure 5:**
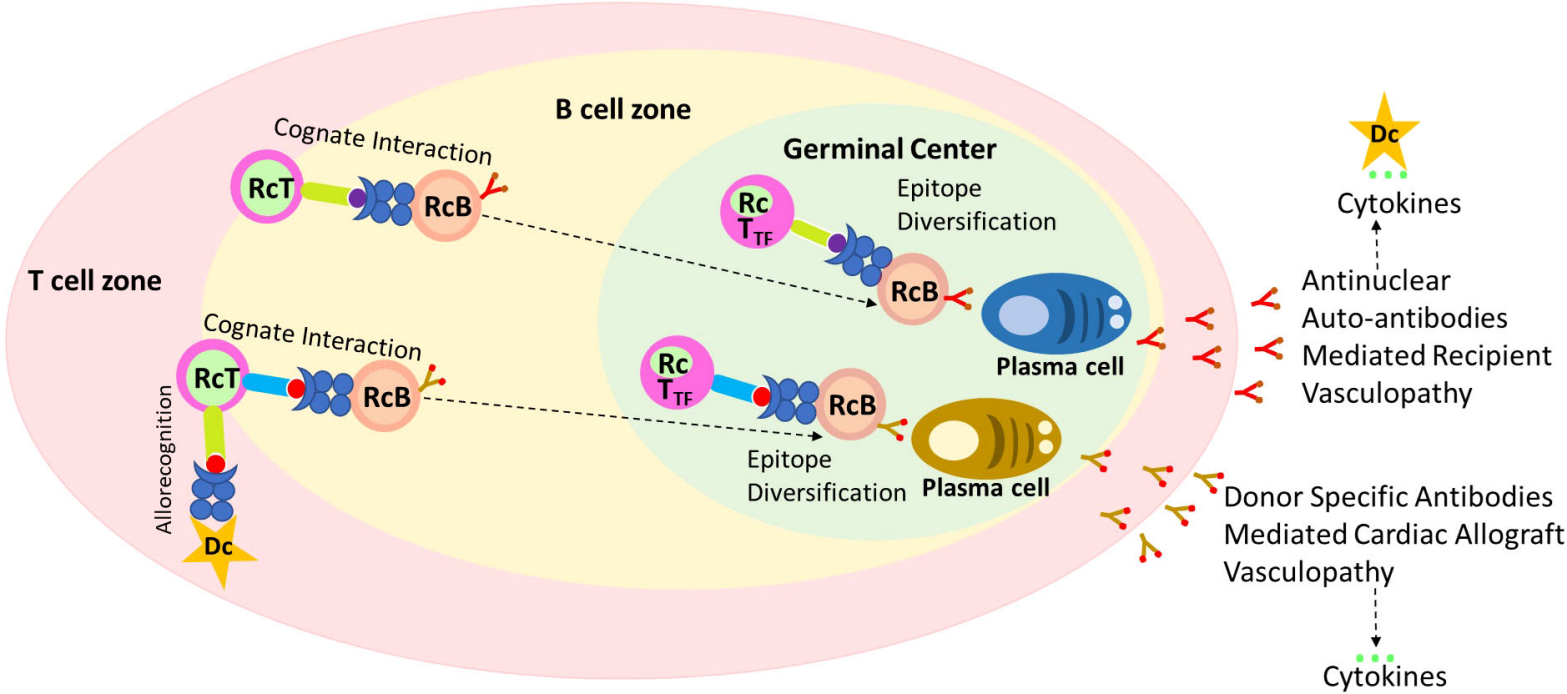
A possible mechanism of ANA to trigger the development of IgG *de novo* DSA. Recognition of antigenic peptides by T cell leads to the migration of B cell to the T:B cell border. Due to defects in ARE genes in some recipient T cells recognize self-peptide as non-self-loaded on the B cells, and upon their cognate interaction these cells become activated. Licensed recipient T cells travel with the B cell to form long-lasting autoreactive germinal centers, which support late epitope diversification to embrace, among other target antigens, anti-nuclear autoantibody responses. Late germinal center reaction may mediate and cause the development of allograft vasculopathy independently of other effector mechanisms, and results in the cytokine storm. The Proinflammatory cytokines activate antigen presenting cells such as dendritic and B cells leading enhanced antigen presentations against mismatch donor antigens. Licensed T cells along with B cells now form alloreactive germinal center leading to epitope diversification (IgG Class switching), and plasma cell formation, and secretion dnDSA which may cause antibody mediated injury to allograft, and cytokine storm and cycle keep repeating to cause allograft injury.

Researchers have a vested interest in both non-HLA immunity and HLA immunity in heart transplantation (4, 8, 9, 12, 21, 22). Anti-HLA IgG DSA is known to activate endothelial cells, resulting in proliferation, cytokine production and release, and recruitment of leukocytes (31, 32). The mechanisms by which non-HLA antibodies contribute to graft injury have not been extensively explored. Non-HLA antibodies contributing to allograft injury are of two types: first, the alloantibodies targeting the mismatched donor non-HLA antigens such as MICA (5), and second, the antibodies that target self-antigens referred to as autoantibodies (4, 6). Accumulating evidence suggests a link between humoral autoimmunity and alloimmunity (3, 7, 33). Similarly, our data indicates a correlation of positive ANA titer, a novel biomarker of autoimmune condition, to predict the development of IgG dnDSA (**Table 3**) and showed the synergistic effect of ANA and IgG dnDSA in ABMR diagnosis (**Table 3**). To provide a possible explanation for our findings, we postulated that an underlying humoral autoimmune condition could trigger or contribute to the development of IgG dnDSA directed against the mismatched donor HLA antigens and, thus, either initiate the development of *de novo* alloimmune response or could synergistically work with *de novo* alloimmune response to initiate the development of antibody mediated rejection in cardiac transplantation (**Figure 5**).

The findings of this study could be useful in stratifying the heart transplant recipients in low, intermediate, and high immunological risk categories for ABMR based on positive anti-nuclear antibodies and presence of circulating IgG dnDSA. Patients with pre-existing non-HLA anti-nuclear antibodies may require closer immunological monitoring and possibly augmented immunosuppression. The standard protocol requires serologic and histopathological studies to confirm ABMR diagnosis in Banff (23) and ISHLT classifications for heart transplantation (24). Modulation of baseline immunosuppression in ANA-positive heart transplant recipients might reduce dnDSA risk. Routine monitoring of IgG dnDSA can enable early interventions, such as optimization of immunosuppression or plasmapheresis in high-risk patients. C1q testing could be utilized to differentiate aggressive dnDSA from bystanders DSA, i.e., complement binding dnDSA from non-complement binding dnDSA. Persistent or increased intensity dnDSA/C1q positive dnDSA may guide therapy intensification. Patients with transient DSAs may not require aggressive intervention but still need close follow-up and could be potentially managed with an augmented dose of immunosuppression.

The present study has some limitations. We did not screen sera for other known deleterious non-HLA antibodies associated with poor heart transplant outcomes, such as anti-vimentin, anti-MICA, and anti-endothelial antibodies. Therefore, we were unable to simultaneously determine the role of these non-HLA antibodies in the context of this study. Due to the retrospective nature of this study, we lack the dataset about non-adherence or compliance with the immunosuppression regimen among the study participants; hence, the impact of non-adherence with the immunosuppression in the development of ABMR in our study participants cannot be precisely described.

In summary, this study establishes that circulating IgG dnDSA is an independent biomarker for ABMR diagnosis in heart transplantation and further refining and confirms the previously known correlation of IgG dnDSA with ABMR. Subsequently, our data revealed that circulating IgG dnDSA and non-HLA antinuclear antibodies have synergistic effects that cause antibody-mediated rejection in heart transplantation. This is a single study center with a retrospective design. Additional studies should be undertaken to evaluate the correlation between ANA titer and IgG dnDSA in ABMR diagnosis and to establish the clinical utility of positive ANA titers in risk stratification for the development of IgG dnDSA in heart transplantation.

## Data Availability

The original contributions presented in the study are included in the article/supplementary material. Further inquiries can be directed to the corresponding author.
